# Chicken Heat Shock Protein 70 Is an Essential Host Protein for Infectious Bursal Disease Virus Infection In Vitro

**DOI:** 10.3390/pathogens10060664

**Published:** 2021-05-28

**Authors:** Yufang Meng, Xiaoxue Yu, Chunxue You, Wenjuan Zhang, Yingfeng Sun, Liuan Li, Tianming Jin, Pengyu Pan, Ailing Xie

**Affiliations:** Tianjin Key Laboratory of Agricultural Animal Breeding and Healthy Husbandry, College of Animal Science and Veterinary Medicine, Tianjin Agricultural University, Tianjin 300392, China; 1803020108@stu.tjau.edu.cn (Y.M.); youchunxue@mail.bnu.edu.cn (C.Y.); zwj0729@mail.bnu.edu.cn (W.Z.); yfsun@tjau.edu.cn (Y.S.); liliuan@tjau.edu.cn (L.L.); jtm680@tjau.edu.cn (T.J.); 1903040116@stu.tjau.edu.cn (P.P.); 2003020147@stu.tjau.edu.cn (A.X.)

**Keywords:** IBDV, Hsp70, dsRNA, replication, host-virus interaction

## Abstract

Infectious bursal disease virus (IBDV) infection causes pathogenicity and mortality in chickens, leading to huge economic losses in the poultry industry worldwide. Studies of host-virus interaction can help us to better understand the viral pathogenicity. As a highly conservative host factor, heat shock protein 70 (Hsp70) is observed to be involved in numerous viral infections. However, there is little information about the role of chicken Hsp70 (cHsp70) in IBDV infection. In the present study, the increased expression of cHsp70 was observed during IBDV-infected DF-1 cells. Further studies revealed that Hsp70 had similar locations with the viral double-stranded RNA (dsRNA), and the result of pull-down assay showed the direct interaction between cHsp70 with dsRNA, viral proteins (vp)2 and 3, indicating that maybe cHsp70 participates in the formation of the replication and transcription complex. Furthermore, overexpression of cHsp70 promoted IBDV production and knockdown of cHsp70 using small interfering RNAs (siRNA) and reducedviral production, implying the necessity of cHsp70 in IBDV infection. These results reveal that cHsp70 is essential for IBDV infection in DF-1 cells, suggesting that targeting cHsp70 may be applied as an antiviral strategy.

## 1. Introduction

Infectious bursal disease (IBD), caused by infectious bursal disease virus (IBDV), is a very acute and highly contagious disease which leads to great loss in poultry. IBDV is a double-stranded RNA virus which belongs to family Birnaviridae [1]. There are two serotypes of IBDV. Serotype 1 strains can cause pathogenicity and mortality in chickens and serotype 2 strains are avirulent to chickens [2,3,4]. IBDV targets chicken B lymphocytes primarily, disrupts the function of the bursa of Fabricius, leads to immunosuppression and makes chickens susceptible to other pathogens. Serotype 1 strains of IBDV are widely propagated in chicken embryo fibroblast cells, such as DF-1 cells and chicken embryo fibroblasts (CEF) [5]. The IBDV genome consists of two segments, segment A encoding vp2, 3, 4, 5 and segment B encoding vp1 [6]. vp2 and vp3 are structural proteins of IBDV particles, and the binding between vp2 or vp3 with dsRNA is very important to maintain the stability of virions [7]. As parasitic organisms, many processes in the viral propagation rely on host factors to facilitate, such as adsorption, stripping, genome replication and transcription, viral protein synthesis, virus particle packaging and so on [8,9,10,11,12]. Meanwhile, the defense mechanisms of the host cells are activated to combat the viral infection, such as the innate immune response [13]. Hence, host factors may play positive or negative roles during viral infection. Thus, study of virus–host protein interactions is critical for understanding viral pathogenesis and development of antiviral drugs. 

Heat shock proteins (Hsps) are evolutionarily conserved proteins. The expression of Hsps, including Hsp70, increases following stress exposure, such as heat shock and pathogenic infection. Hsp70 is found highly expressed in many virus infections and is involved in virus entry, replication of the virus genome, modulating virus polymerase activity [14], packaging of virions, and innate immune response of host [15]. Inhibition of Hsp70 reduces the replication and production of reproductive and respiratory syndrome virus (PPRSV) [16], inhibits the EB virus infection [17], decreases the virus titer of Zika virus [18,19] and affects the assembly of hepatitis C virions [20]. Previous studies showed that chicken Hsp90 which belongs to the Hsp family of proteins is involved in IBDV infection. cHsp90 participates in IBDV entry into DF-1 cells through interaction with subviral particles which are formed from vp2 [10]. Application of anti-cHsp90 microRNAs could inhibit IBDV infection and furthermore cHsp90α, not cHsp90β, functions in IBDV infection [21]. Heat conditioning causes increased Hsp70 expression and a decreased bursal histological score during IBDV infection [22]. A DNA vaccine developed using a full length vp2 gene of IBDV fused with truncated Hsp70 of *Mycobacterium tuberculosis* could provide very good protection against IBDV [23]. Heat shock cognate protein 70 (HSC70), a member of the Hsp70 family, is required for IBDV infection by interacting with vp2 [24]. Results from previous studies indicate that Hsp70 should be involved in IBDV infection. However, the exact roles of Hsp70 in IBDV infection have not been evaluated. 

In this research, we aimed to reveal whether cHsp70 is involved in IBDV infection. We observed that the expression of cHsp70 increased in IBDV-infected DF-1 cells, and siRNA interference targeting cHsp70 lead to lower production of IBDV. Furthermore, we observed the direct interaction of Hsp70 and dsRNA of IBDV. Overexpression of cHsp70 promoted viral production and siRNA towards cHsp70 weakened viral production. These results indicate that chicken Hsp70 plays a positive role in DF-1 cells during IBDV infection. In this study, for the first time the interaction between host protein-Hsp70 and IBDV is revealed, which would provide a new sight into IBDV pathogenesis.

## 2. Results

### 2.1. Viral Load Changes in IBDV-Infected DF-1 Cells

**Figure 1:**
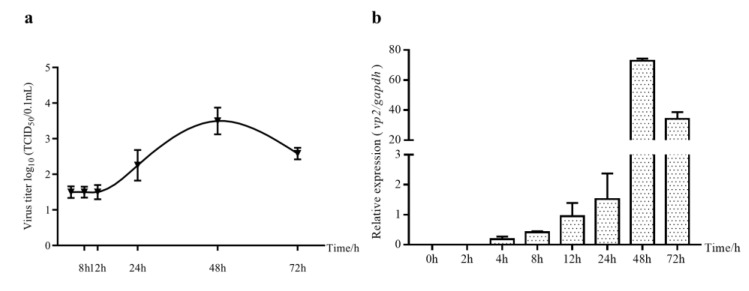
The viral load in IBDV-infected DF-1 cells at different times (MOI = 1): (**a**) TCID_50_ was measured by IFA. (**b**) The expression of the *vp2* gene by qRT-PCR.

DF-1 cells are susceptible to Ts strain IBDV and are used for propagating this strain in our laboratory routinely. To better understand the reproduction characteristics of IBDV in DF-1 cells, virus titers by TCID_50_ and vp2 expression by quantitative real-time PCR (qRT-PCR) were detected at different times post IBDV infection. As Figure 1a showed, the virus titers changed just after 12 h post infection (hpi), increasing rapidly from 24 hpi, peaking at 48 hpi and then decreasing. The trend was consistent with vp2 expression (Figure 1b).

### 2.2. The Expression of cHsp70 Gradually Increased during IBDV Infection

**Figure 2:**
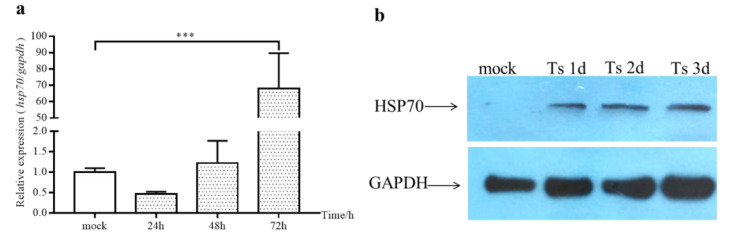
The expression of cHsp70 at mRNA and protein levels increased during Ts infection. (**a**) Changes of cHsp70 expression at mRNA level detected by qRT-PCR. *p* ≤ 0.001 (***). (**b**) Changes of cHsp70 expression at protein levels detected by Western blot.

To test whether Hsp70 is associated with IBDV infection, we detected the expressions of cHsp70 mRNA and cHsp70 protein in DF-1 cells during infection of Ts strain with IBDV. The expression of cHsp70 of IBDV-infected cells at 72 hpi was more than 60-fold that of the mock group (Figure 2a). We also observed the increased expression of the Hsp70 protein along with IBDV infection (Figure 2b). These results indicated that Hsp70 is involved in IBDV infection.

### 2.3. cHsp70 has Direct Interaction with IBDV in DF-1 Cells

**Figure 3:**
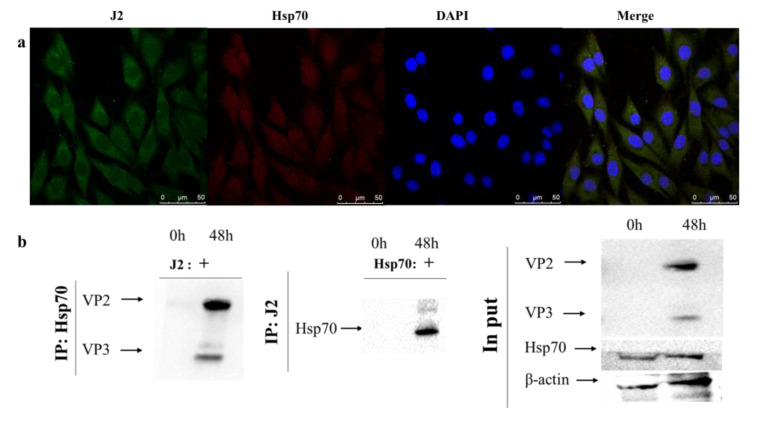
Direct interaction between Hsp70 and dsRNA. (**a**) Location of Hsp70 and dsRNA after IBDV infection; (**b**) Western blot results after pull-down assay. Left: anti-Hsp70 antibodies-pulling out proteins from mock-infected and IBDV-infected DF-1 cells. Middle: J2-pulling out proteins from mock-infected and IBDV-infected DF-1 cells. Right: total proteins from mock-infected and IBDV-infected DF-1 cells.

To further verify the relationship between cHsp70 and viral dsRNA genome or viral proteins, confocal immunofluorescence microscopy and pull-down assay were conducted to detect cHsp70 and dsRNA. Our results showed that cHsp70 was detected to colocalize with IBDV dsRNA in the cytoplasm of IBDV-infected DF-1 cells (Figure 3a). Pull-down assay showed the direct interaction of cHsp70 and dsRNA (Figure 3b). Additionally, VP2 and VP3 were detected in cHsp70-pulled out proteins because these two special proteins are pulled out by the antibodies for dsRNA (J2 antibody) (Figure 3b). These results revealed that the interaction between cHsp70 and dsRNA of IBDV was direct. The replication and transcription complex (RTC), which contain viral and host proteins and dsRNA, was generated during infection of RNA virus. The direct interaction between cHsp70, dsRNA and viral proteins indicated that perhaps cHsp70 is involved in the formation of RTC of IBDV.

### 2.4. Overexpression of cHsp70 Promotes the Production of IBDV

**Figure 4:**
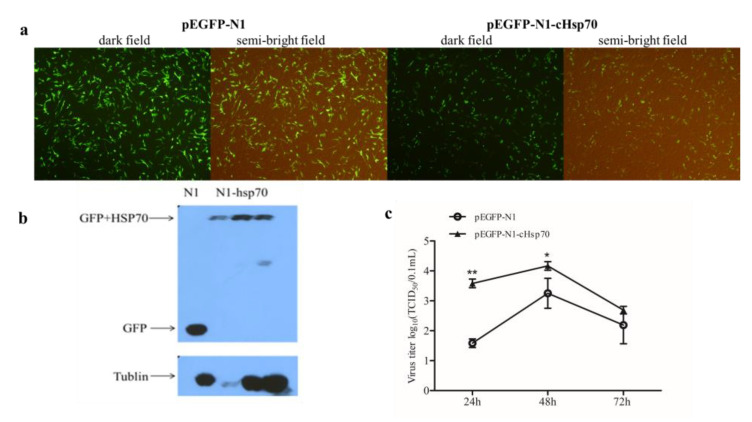
Overexpression of cHsp70 promotes the production of IBDV. (**a**) The transfection of pEGFP-N1 and pEGFP-N1-cHsp70 into DF-1 cells was observed under a fluorescence microscope. (**b**) Western blot results for detecting overexpression of cHsp70 proteins (from left to right, where three lanes of pEGFP-N1-cHsp70 transfected DF-1 cells means different time points post transfection): 24, 48, 72 h. (**c**) The changes of viral titers after cHsp70 were overexpressed at 24, 48, 72 h post IBDV infection. Differences were regarded as significant at *p* ≤ 0.05 (*) and very significant at *p* ≤ 0.01 (**).

To determine whether cHsp70 plays a positive or negative role in IBDV infection, overexpression of cHsp70 in DF-1 cells was conducted and viral titers were analyzed. The full length of the cHsp70 gene was cloned and attached to the eukaryotic expression vector pEGFP-N1. Then pEGFP-N1 and pEGFP-N1-cHsp70 were transfected into DF-1 cells. As Figure 4a,b show, cHsp70 was successfully overexpressed in DF-1 cells.

Next, the effects of cHsp70 overexpression on viral titers were evaluated by TCID_50_. Results showed that the viral titer significantly increased in cHsp70 overexpressed DF-1 cells, suggesting that cHsp70 promotes the production of IBDV (Figure 4c). These results showed that cHsp70 plays a positive role in IBDV infection.

### 2.5. Inhibition of cHsp70 by siRNA Attenuates the Production of IBDV

**Figure 5:**
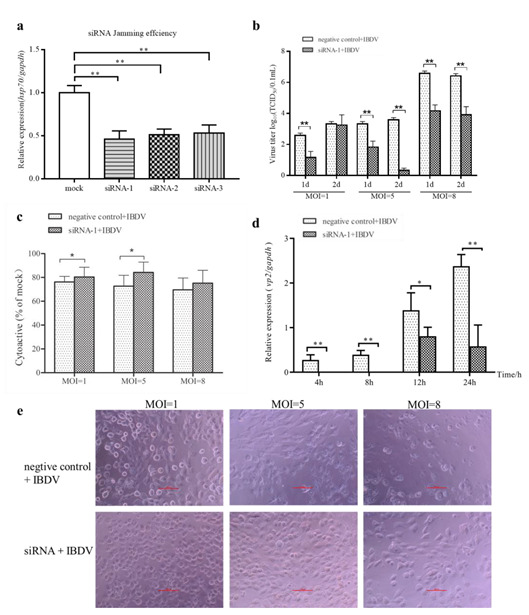
Inhibition of cHsp70 by siRNA attenuates the production of IBDV. (**a**) Interference efficiencies of siRNAs targeting cHsp70 in DF-1 cells were calculated by qRT-PCR. (**b**) The changes of viral titers after siRNA was added. (**c**) The cell viability detected by CCK-8 assay. (**d**) The changes in *vp2* gene expression when siRNA interference was carried out. (**e**) Comparison of the cytopathy effects by the addition of siRNA by visual study when CCK-8 assay was conducted. Differences were regarded as significant at *p* ≤ 0.05 (*) and very significant at *p* ≤ 0.01 (**).

To further confirm whether cHsp70 is an essential host protein in IBDV infection, siRNAs were used to evaluate the necessity of cHsp70 in IBDV infection of DF-1 cells. As Figure 5a shows, all three siRNAs significantly reduced the expression of cHsp70 in DF-1 cells. The siRNA that interfered most efficiently (siRNA-1) was chosen for follow-up experiments. 

Then we evaluated the effects of siRNA-1 on viral titers by TCID_50_. Results showed that siRNA-1 decreased the viral titer significantly, suggesting that inhibition of cHsp70 led to the reduced production of IBDV (Figure 5b). Additionally, the result of the CCK-8 assay displayed that in the siRNA-1 interferential group, the cell viability was higher than that of the IBDV-infected group (Figure 5c), and the cytopathy effect caused by IBDV was significantly alleviated by the addition of siRNA via visual study (Figure 5e). As Figure 5d showed, *vp2* gene expressions was inhibited by siRNA-1 interference. Furthermore, *vp2* gene expression of the siRNA-interference group at 4 and 8 h post IBDV infection was not detected (Figure 5d). These results showed that cHsp70 is essential for IBDV infection.

## 3. Discussion

IBD leads to great economic losses in the poultry industry worldwide. So far, the only way to combat this highly contagious viral infection is with vaccination. Identification of host factors during IBDV infection can provide a greater insight into the virus pathogenesis and a potential for development of antiviral strategies. 

Although the B lymphocyte of the bursa of Fabricius is the target of IBDV, DF-1 cells are widely used for the propagation of IBDV. The DF-1 cell line is derived from chicken embryos and develops into a spontaneously immortalized line of CEF, which is widely used in studies regarding pathogenicity of avian viruses [25]. The Ts strain of IBDV was first isolated from the Tianshui region of Gansu province in China in 1992, and then passed through CEF cells to adapt to the cells. In this study, we detected virus titers or *vp2* expressions in Ts-infected DF-1 cells at different times of infection. *vp2* is a major viral protein of the IBDV virion, hence *vp2* gene expression is regarded as an indicator of viral load in host cells. In general, the titer increased after 12 hpi and peaked at 48 hpi, which is consistent with our previous study [26].

Virus infection changes the expressions of many host proteins. In this study, we observed that IBDV infection induced cHsp70 expression at the mRNA level and protein level in DF-1 cells (Figure 2), indicating that cHsp70 may play a role in IBDV infection. There were significantly more cHsp70 proteins present in IBDV-infected DF-1 cells at 24, 48 and 72 hpi than in mock group, however the expression of *cHsp70* mRNA at 24 and 48 hpi was not higher than in mock group. Conventionally, protein levels are primarily determined by mRNAs or by delayed synthesis which occurs between mRNA and proteins [27]. The phenomenon of low mRNA level but high protein level may be due to the short half-life period of mRNA or the existence of polyribosomes which could promote protein synthesis efficiency. However, more experimental evidence is needed to test this conjecture.

The role of Hsp70 in viral infection might be positive or negative. Hsp70 has a proviral function during rabies virus infection [28] and porcine circovirus type 2 [29]. However, Hsp70 negatively controls the bioavailability of viral proteins in rotavirus infection [30]. To better determine the role of cHsp70 in IBDV infection, we detected the viral titer when cHsp70 was overexpressed by transfection of recombinant pEGFP-N1-cHsp70. Overexpression of cHsp70 in DF-1 cells increased IBDV titer, indicating that cHsp70 plays a positive role in IBDV infection (Figure 4). To explore whether cHsp70 is essential in IBDV infection, cHsp70 expression was reduced by siRNA. To avoid the inhibitory effects of commercially available heat shock protein inhibitors on other proteins, siRNA specific to cHsp70 was designed and synthesized. The highest interference efficiency of siRNA designed in this study was 54%. Results showed that knockdown of cHsp70 led to inhibition of viral production. The results indicate that cHsp70 plays a positive role in IBDV infection.

Entry of IBDV into cells proceeds through *vp3*, cell receptors, Fc receptors and endocytosis. Endocytic vesicles then bind to lysosomes. After partial degradation of the virions, the capsid is removed, and the dsRNA genome is released to continue replication and transcription [31]. RTC were generated during RNA virus infection which contain viral and host proteins and dsRNA. Many researchers regarded dsRNA as a marker of RTC in cells infected with RNA viruses [32,33,34]. To investigate whether HSP70 is involved in IBDV replication, we detected the dsRNA level using specific antibody-J2 antibody. The results showed that cHsp70 and dsRNA in IBDV-infected DF-1cells had similar colocalization and directly interact (Figure 3), indicating that HSP70, *vp2* and *vp3* are involved in the formation of RTC of IBDV. We observed that the expression of the *vp2* gene could not be detected at 4, 8 hpi when siRNA was added (Figure 5d). The results indicate that the role of cHsp70 in IBDV infection is not limited to participating in IBDV replication but also to virus entry. However, this conjecture needs more experimental evidence.

In summary, our study revealed cHsp70 plays a positive role in IBDV infection by directly interacting with IBDV dsRNA, indicating that targeting cHsp70 may be a way to fight against the virus.

## 4. Materials and Methods

### 4.1. Cells and Viruses

DF-1 cell line and cell adapted strain-Ts strain IBDV were both preserved in our laboratory. DF-1 cells were grown in Dulbecco’s modified Eagle’s medium (DMEM) containing 10% fetal bovine serum (FBS) and 1% penicillin-streptomycin. Ts strain IBDV was propagated in DF-1 cells using DMEM supplemented with 2% FBS. 

### 4.2. Chemicals, Reagents, Antibodies and Kits

FBS and DMEM were purchased from Gibco Inc., Carlsbad, CA, USA; FITC-coupling anti-chicken IgY antibody was purchased from Southern Biotechnology Associates Inc.; rabbit anti-HSP70 antibody was purchased from Abcam Inc.(Cambridge, UK); J2 antibodies was purchased from Scicons Inc. (Szirák, Hungary); Cy3-coupling anti-rabbit IgG secondary antibodies were purchased from Beyotime Inc. (Shanghai, China); DAPI was purchased from Solarbio Inc. (Beijing, China); prefabricated SDS-PAGE gel was purchased from GenScript Inc. (Nanjing, China); anti-mouse IgG secondary antibody conjugated with HRP was purchased from CWBIO Inc. (Beijing, China); SuperSignal West Pico PLUS Chemiluminescent substrate was purchased from Thermo scientific Inc.(Rockford, IL, USA); siRNAs were designed and synthesized by GenePharma Inc. (Shanghai, China); total RNA extraction kit was purchased from Tiangen Biotechnology Co. (Beijing, China); Gel extraction kit was purchased from Axygen (San Francisco, CA, USA); Seamless cloning and assembly kit was purchased from Novoprotein Inc. (Shanghai, China); GoScript reverse transcription kit was purchased from Promega (Madison, WI, USA); all-in-one qRT-PCR Mix SYBR Green I Master Mix was purchased from GeneCopoeia Inc. (Baltimore, MD, USA); CCK-8 assay kit was purchased from G-clone Biotechnology Co., Ltd. (Beijing, China).

### 4.3. Determination of 50% Tissue Culture Infectious Dose (TCID_50_) by IFA

DF-1 monolayer cells were prepared in 96-well cell culture plate. The culture medium was discarded, and the cells were washed with phosphate buffer saline (PBS) three times. Cells were then inoculated with 100 μL of continuous ten-fold virus dilution. Each dilution degree had three duplicate wells. IFA was performed t 4 to 6 days post infection and 4% paraformaldehyde fixation of cells were washed with PBS three times and drilled with 1% Triton X-100 for 30 min. DF-1 cells were incubated with anti-IBDV chicken serum and FITC-coupling anti-chicken IgY antibody in succession. Fluorescence intensity was observed under a fluorescence microscope and TCID_50_ was calculated according to the Reed–Muench formula.

### 4.4. Location Detection by IFA

DF-1 cells were prepared in a 24-well plate in monolayers and were infected with Ts strain IBDV for 48 h. Fixation and drilling of DF-1 cells were conducted as 4.2. Cells were incubated with rabbit anti-Hsp70 antibodies diluted with 2% BSA (1:100), J2 antibodies and Cy3-coupling anti-rabbit IgG secondary antibodies consecutively. Finally, the nucleus was stained with DAPI and the cells were observed under a fluorescence microscope.

### 4.5. Co-Immunoprecipitation (Co-IP) Assay

DF-1 monolayer cells were infected with Ts strain IBDV (MOI = 1). At 48 h post infection, cells were washed with precooling PBS, scraped off with a cell scraper in pre-cooled RIPA cracking buffer, transferred to a clean 1.5 mL EP tube and remained static cracking for 30 min. After centrifugation at 14,000 g at 4 °C for 15 min, the supernatant was immediately transferred to a new EP tube. The concentration of total protein was determined by NanoDrop One (Thermo Fisher scientific, Madison, WI, USA) and then divided into two equal volumes. The same two protein samples were incubated with 1 μg J2 antibodies or 1 μg anti-Hsp70 antibodies, respectively. Then, 40 μg protein A agarose was added to the tubes, and the tubes were put on low speed and shaken overnight at 4 °C. After centrifugation at 14,000 g for 5 s, the precipitation was collected and washed with pre-cooled washing buffer three times. The supernatant was removed and 40 μL of SDS-PAGE loading buffer was added and boiled for 10 min.

### 4.6. Western Blot

SDS-PAGE were performed as described previously [26]. Samples were loaded onto a prefabricated gel and electrophoresis was conducted at a voltage of 120 V. Following electrophoresis, the gel was used for Western blot analysis by electroblotting onto a polyvinylidene difluoride (PVDF) membrane. 

To detect IBDV antigens (dsRNA), the J2 antibodies were employed as the primary antibody. To detect cHsp70, anti-Hsp70 antibody was used as the primary antibody. To detect the overexpression of cHsp70, anti-GFP antibody was used as the primary antibody. β-actin and tubulin was used as an internal reference. Following washing, the membrane was incubated with the appropriate secondary antibody conjugated with HRP, and the bands were observed after adding chemiluminescent substrate.

### 4.7. Overexpression of cHsp70 in DF-1 Cells

The full length of the cHsp70 gene with a homologous arm of pEGFP-N1 was amplificated from chicken thymus cDNA. The sequences of the primer are shown in Table 1.

The full length of the cHsp70 gene and pre-enzymatic cleaved vector were extracted using a gel extraction kit and then seamless cloning was done to develop pEGFP-N1-cHsp70. pEGFP-N1-cHsp70 was transfected into DF-1 cells in a 24-well cell culture plate following the instructions of Liposome3000. Twenty-four hours post transfection, protein samples were collected to detect the overexpression by Western blot or IBDV infection was conducted. After 24 h of IBDV infection, the supernatant was collected by centrifugation after freeze-thaw three times as the samples to test TCID_50_.

### 4.8. siRNA Interference

The sequences of siRNAs we designed and synthesized are shown in Table 2. DF-1 cells were transfected with 20 μM of siRNA following the instruction of Lipidosome3000. Twenty-four hours post transfection, total RNA was collected with a total RNA extraction kit followed by reverse transcription PCR into cDNA. The interference efficiencies of siRNAs were detected by real-time fluorescence quantitative PCR (qRT-PCR). 

### 4.9. Cell Activity Detection by CCK8 Assay

DF-1 monolayer cells were prepared in a 96-well cell culture plate. siRNA was transfected into cells, and 24 h later the cells were infected by IBDV with MOI = 1. After 24 h, 10 μL of CCK-8 solution was added to each well. The plate was incubated for 1–4 h. The absorbance at 450 nm was measured with the enzyme plate instrument.

Cell viability (%) = [A(dosing) − A(blank)]/[A(0 dosing) − A(blank)]×100

A (dosing): absorbance of the hole with cells, CCK-8 solution and drug solution (siRNA transfection or IBDV infection); A (blank): absorbance of the hole with medium CCK-8 solution without cells;

A (0 dosing): absorbance of the hole with cells, CCK-8 solution and no drug solution.

### 4.10. qRT-PCR Analysis of Gene Expression

The concentration of the extracted total RNA was detected by NanogDrop one. Subsequently, 1 μg of RNA was inverse transcribed into cDNA using GoScript reverse transcription kit (Promega, WI, USA). 

qRT-PCR was performed in 15 μL of GeneCopoeia all-in-one qPCR Mix SYBR Green I Master Mix with the (Bio-Rad Protocol) Real-Time PCR System. The individual primers used are shown in Table 3. The cycling parameters were as follows: 95 °C, 10 min; 40 cycles of 95 °C, 15 s and 60 °C, 1 min; 65 °C, 5 s, 95 °C, 5 min. The gene expressions were calculated relative to the expression of the reference gene, glyceraldehyde 3-phosphate dehydrogenase (*gapdh*). Increases or decreases relative to the untreated samples were expressed as fold changes, which were calculated using Bio-Rad CFX Manager.exe 2.1.

### 4.11. Data Analysis

Statistical analyses were performed with the Statistical Package for the Social Sciences (SPSS) version 20.1 software (SPSS Inc., Chicago, IL, USA). Independent-samples *t*-tests were used to test for significant differences. Differences were regarded as significant at *p* ≤ 0.05 (*) and very significant at *p* ≤ 0.01 (**).

## 5. Conclusions

In conclusion, this is the first time the direct interaction of chicken Hsp70 with IBDV infection in vitro is revealed. IBDV infection induced the expression of cHsp70 and knockdown of cHsp70 reduced viral production, indicating that cHsp70 plays a positive role in IBDV infection. These findings help us better understand the pathogenicity of IBDV and provide good insight for combating the disease.

## Figures and Tables

**Table 1:** Sequences of the primer for amplification of cHsp70.

Name.	Direction	Sequence
pEGFP-N1-cHsp70	Forward	ATT CTG CAG TCG ACG GTA C ATGTCTGGCAAAGGGCCG
	Reverse	GAC CGG TGG ATC CCG GGCATCTACTTCTTCAATGGTTG

**Table 2:** Sequences of siRNAs.

Name	Direction	Sequence
siRNA-1	Forward	CCCGCUUACUUCAACGACUTT
	Reverse	AGUCGUUGAAGUAAGCGGGTT
siRNA-2	Forward	GCGUGACAAUGCUGGCAAUTT
	Reverse	AUUGCCAGCAUUGUCACGCTT
siRNA-3	Forward	GCAAGCCAGCAUUGAGAUUTT
	Reverse	AAUCUCAAUGCUGGCUUGCTT

**Table 3:** Sequences of the primers used in qRT-PCR.

Gene	Direction	Sequence
cHsp70	Forward	TGTTATCACAGTGCCCGCTTAC
	Reverse	CACGTTAAGGCCAGTGATGG
cGAPDH ^a^	Forward	CTCTGCCCCCTCTGCTGAT
	Reverse	CAGGAGGCATTGCTGATGATC
T_S_^b^	Forward	ACCGGCACCGACAACCTTA
	Reverse	CCCTGCCTGACCACCACTT

^a,b^ Primer from Xiaoxue Yu et al., 2015 [26].

## Data Availability

The data presented in this study are available within this article.
